# High-Temperature Chlorination of Nickel Oxide Using Calcium Chloride

**DOI:** 10.3390/ma16216888

**Published:** 2023-10-27

**Authors:** Peiwei Han, Jingmin Yan, Lunliang Zhang, Zhengchen Li, Shufeng Ye

**Affiliations:** 1 State Key Laboratory of Multiphase Complex Systems, Institute of Process Engineering, Chinese Academy of Sciences, Beijing 100190, China; 18813095233@163.com (J.Y.); zll15879704740@163.com (L.Z.); lizhengchen19@ipe.ac.cn (Z.L.); 2 Innovation Academy for Green Manufacture, Chinese Academy of Sciences, Beijing 100190, China; 3 University of Chinese Academy of Sciences, Beijing 100049, China

**Keywords:** chlorination-volatilization, nickel oxide, calcium chloride

## Abstract

Attempts have been made to extract nickel from ores and nickel-containing wastes using the chlorination method. However, the use of gaseous chlorinating agents is limited due to their toxicity. High-temperature chlorination of nickel oxide using calcium chloride is analyzed in this study. The volatilization percentage is positively correlated to temperature and CaCl_2_ dosage and negatively correlated to oxygen partial pressure. The apparent activation energy is calculated to be 142.91 kJ/mol, between 1173 K and 1323 K, which suggests that the high-temperature chlorination of nickel oxide using calcium chloride is controlled by a chemical reaction.

## 1. Introduction

Nickel is widely used for the manufacturing of special steels, special alloys, batteries, and catalysts. Attempts have been made to extract nickel from ores and nickel-containing wastes using the chlorination method in which nickel oxide is converted into soluble or gaseous nickel chloride when roasted in the presence of chlorinating agents [1,2,3,4,5,6,7,8,9,10,11,12,13,14,15].

Selective chlorination was carried out to recover valuable metals from spent catalysts in a series of studies by Gaballah et al. [1,2,3]. The spent catalysts were roasted with Cl_2_-N_2_ [1,2,3], Cl_2_-O_2_ [1], Cl_2_-CO [1], Cl_2_-air [1,2,3], Cl_2_-CO-N_2_ [2,3] at a temperature between 473 K and 1173 K. Up to 98% of nickel could be recovered after roasting under the optimum conditions and following a subsequent water leaching.

Alvarez and Bohé [4] investigated the direct chlorination of nickel-containing materials under an Ar-Cl_2_ atmosphere. They found that the beginning temperatures of the chlorination reaction were 998 K, 746 K, and 701 K for NiO, the mixture of NiO and Al_2_O_3_, and the NiO-Al_2_O_3_ catalyst, respectively. The recovery percentages were 85% and 96% from the mixture and catalyst when roasted at 1073 K, respectively.

The beginning temperatures of nickel oxide chlorination were 573 K and 1023 K for chlorine and calcium chloride, respectively [5]. The presence of active additives (C, BaS, S) shifted the equilibrium of the reaction toward the formation of nickel chloride. Similar trends were also obtained for the chlorination of nickel ferrite [6] and nickel silicate [7].

Selective chlorination was also adopted to extract nickel from reduced or unreduced laterite, followed by water or hot-acidulous water leaching [8,9,10]. The chlorination agents included the mixtures of NaCl and MgCl_2_·6H_2_O (mass ratio 0.4) [8], AlCl_3_·6H_2_O [9], and gaseous HCl [10]. The leaching percentage was 87% from laterite after roasting at 1173 K for 90 min with about 19 wt% of mixtures of NaCl and MgCl_2_·6H_2_O (mass ratio 0.4) as an addition [8]. The contact chance between reactants and ores increases because the eutectic mixtures of NaCl and MgCl_2_·6H_2_O easily penetrate into tiny pore. A total of 91% of Ni was extracted from reduced limonitic laterite (goethite FeOOH and hematite Fe_2_O_3_) using water leaching at 353 K, after roasting at 733 K for 120 min with 40 wt% of AlCl_3_·6H_2_O in addition [9]. To further understand the process, selective chlorination of pre-reduced limonitic laterite was conducted in a HCl-O_2_-H_2_O-N_2_ atmosphere to investigate the kinetics and the effects of temperature, partial pressure of hydrogen chloride, oxygen, water vapor, and total gas-flow rate [10].

Nickel oxide is converted into gaseous chloride at higher temperatures. A thermogravimetric analysis (TGA) technique was used to investigate the chlorination behaviors of nickel oxide in chlorine and hydrogen chloride [11]. TGA curves showed that nickel oxide started to chloridize at 773 K and 673 K in a chlorine atmosphere and a hydrogen chloride atmosphere, respectively, and nickel chloride started to volatilize at 1073 K in both atmospheres. Fruehan and Martonik measured the rate of chlorination of NiO and NiFe_2_O_4_ with Cl_2_ at 1073 to 1473 K and with HCl at 1073 to 1273 K diluted with He or Ar [12]. The rates were affected by mass transfer for all cases. A high-temperature chlorination method was also used for metal recovery from roasted printed-circuit-board waste [13]. The nickel’s volatilization percentage increased with the temperature in chlorine gas; they were both about 85% at 1073 K and 1173 K, respectively.

However, the use of gaseous chlorinating agents is limited due to their toxicity. This study aims to investigate the high-temperature chlorination of nickel oxide using calcium chloride as a chloridizing agent. Effects of different variables on nickel-volatilization percentage and kinetics are investigated, where variables include flow rate, temperature, molar ratio of CaCl_2_ to NiO, and oxygen partial pressure.

## 2. Materials and Methods

Reagent-grade NiO, SiO_2_, Fe_2_O_3_, and anhydrous CaCl_2_ were used in this study. Samples before roasting were prepared according to the composition shown in Table 1. Effects of different variables on nickel-volatilization percentage were investigated with the compositions of Group A to Group C. The composition of Group D was used to study the high-temperature chlorination kinetics of nickel oxide using calcium chloride. The molar ratios of CaCl_2_ to NiO for Group A, B, C, and D were 10, 7.5, 5, and 7.5, respectively, and those of SiO_2_ to CaCl_2_ were 4, 4, 4, and 2, respectively. After sufficient mixing, the mixtures were pressed into briquettes.

The experiment of the effects of different variables were carried out in a horizontal tube furnace. Carrier gas consisted of high-purity nitrogen and oxygen, whose flow rates were controlled with mass-flow controllers. An alumina boat (length: 60 mm, width: 30 mm) with about 10 g of briquettes was located on the center of quartz tube at about 873 K and heated to the desired temperature with a rate of 25 K/min. Samples were cooled inside the furnace to about 873 K after holding them at desired temperatures for 1 h and then cooling to room temperature with the protection of high-purity nitrogen.

The experiments of kinetics were conducted in a muffle furnace under a controlled atmosphere. High-purity nitrogen was injected into the hearth through the inlet below the thermocouple and escaped through the outlet at the top near the door. An alumina boat with about 10 g of briquettes was put at the center of the furnace hearth at desired temperatures. The sample was taken out immediately after holding for a certain time and then cooled to room temperature with the protection of high-purity nitrogen. Exhaust gas was discharged into the atmosphere after alkaline-solution treatment for all the experiments.

Nickel concentrations of samples were determined using an inductively coupled plasma optical emission spectrometer (ICP-OES, Optima 8000, PerkinElmer, Waltham, MA, USA) after dissolution with acid. The nickel-volatilization percentage was calculated using Equation (1).

(1) $$\eta =\frac{c_{i}w_{i}-c_{f}w_{f}}{c_{i}w_{i}}\times 100\%,$$

where *w_i_* and *w_f_* are the sample weights before and after roasting, respectively. *c_i_* and *c_f_* are nickel concentrations before and after roasting, respectively. The collected volatile matters that condensed near the gas outlet were analyzed with X-ray diffraction (XRD, X’PertPro, PANalytical, Almelo, The Netherlands).

## 3. Results and Discussions

### 3.1. Chlorination Product

**Figure 1:**
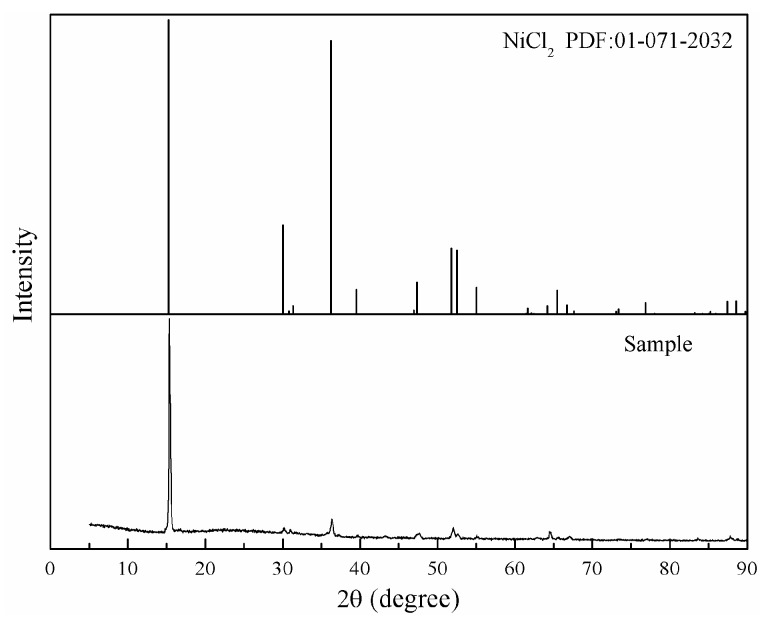
XRD results of collected condensate.

As shown in Figure 1, NiCl_2_ is the main phase of condensed volatile matter. The nickel concentration of condensed volatile matter is 45.12 wt%, which is very close to the nickel’s theoretical concentration of NiCl_2_ (45.29 wt%). It means that NiCl_2_ is the main volatilization product during the high-temperature chlorination of nickel oxide using calcium chloride.

### 3.2. Effect of Gas-Flow Rate

**Figure 2:**
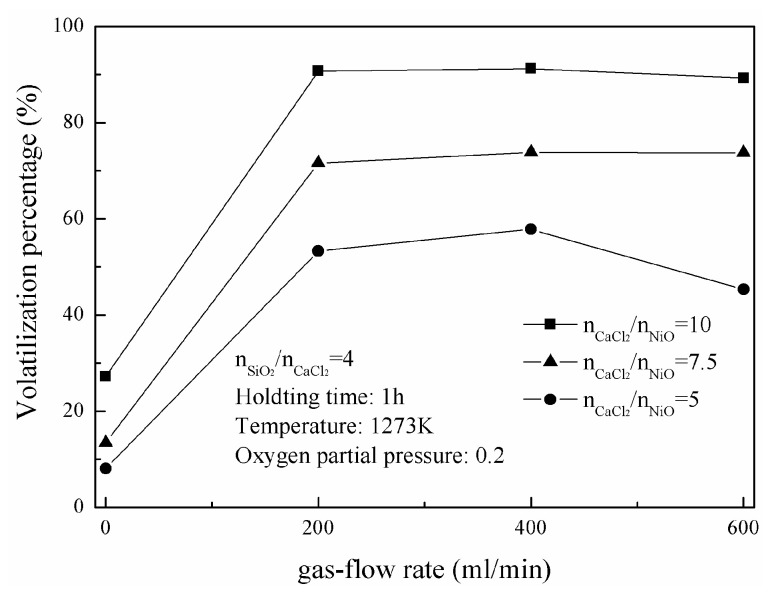
Change of nickel-volatilization percentage with gas-flow rate.

The change of nickel-volatilization percentage with carrier gas-flow rate is shown in Figure 2. Nickel-volatilization percentage becomes larger as gas-flow rate increases from 0 mL/min to 200 mL/min, which could be ascribed to the diffusion strengthening of gaseous reaction products. And, it keeps at a near constant with a larger gas-flow rate when the molar ratios of CaCl_2_ to NiO are 7.5 and 10, respectively. It decreases as gas-flow rate increases from 400 mL/min to 600 mL/min with the molar ratio of five CaCl_2_ to NiO, which could be attributed to the loss of chloridizing agents (gaseous CaCl_2_ or Cl_2_ generated from the decomposition of CaCl_2_). Therefore, it is suitable for all subsequent experiments with the gas flow rate of 400 mL/min to obtain their maximum volatilization percentages.

### 3.3. Effect of CaCl_2_ Dosage

**Figure 3:**
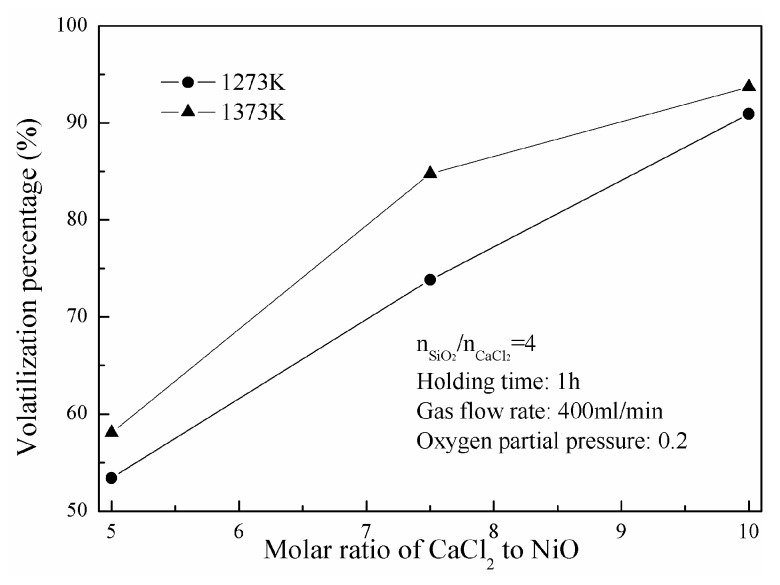
Effect of CaCl_2_ dosage on the nickel-volatilization percentage.

Figure 3 shows the change of nickel-volatilization percentage with different molar ratios of CaCl_2_ to NiO. It increases significantly from 53.4% to 90.9% as the molar ratio of CaCl_2_ to NiO is strengthened from 5 to 10 at 1273 K. A similar trend is observed at 1373 K. It increases from 58.1% to 93.8% as the molar ratio of CaCl_2_ to NiO increases from 5 to 10 at 1373 K.

The direct chlorination of nickel oxide in the presence of SiO_2_ could be written as

CaCl_2_ + SiO_2_ + NiO = NiCl_2_ (g) + CaSiO_3_. (2)

While, the indirect chlorination of nickel oxide in the presence of SiO_2_ could be written as

CaCl_2_ + SiO_2_ + 0.5O_2_ = Cl_2_ + CaSiO_3_, (3)

NiO + Cl_2_ = NiCl_2_ (g) + 0.5O_2_. (4)

The chlorination of nickel oxide is promoted with greater CaCl_2_ dosage which accelerates the balance of high-temperature chlorination turning it to right in the case of direct chlorination. Meanwhile, a greater CaCl_2_ dosage means more Cl_2_ is released with the decomposition of CaCl_2,_ and more nickel oxides are subsequently chlorinated in the case of indirect chlorination. Therefore, it is beneficial to obtain a larger nickel-volatilization percentage with a higher CaCl_2_ dosage.

### 3.4. Effect of Roasting Temperature

**Figure 4:**
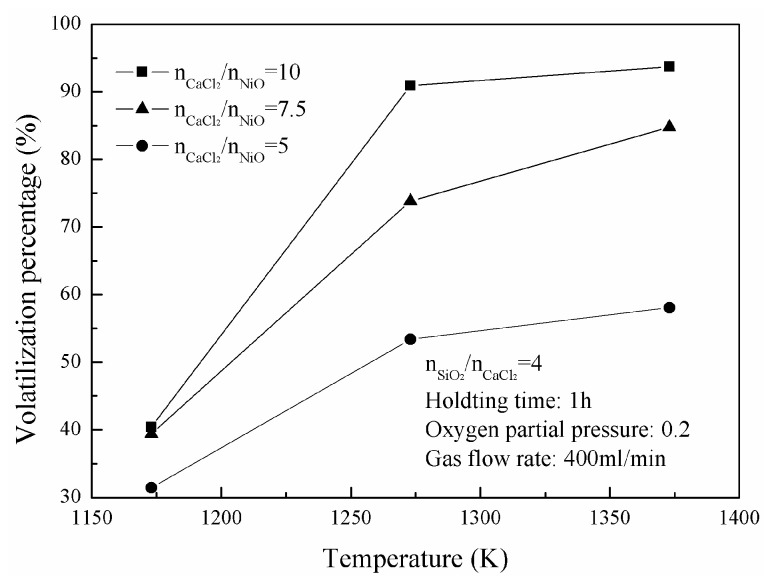
Change of nickel-volatilization percentage with roasting temperature.

**Figure 5:**
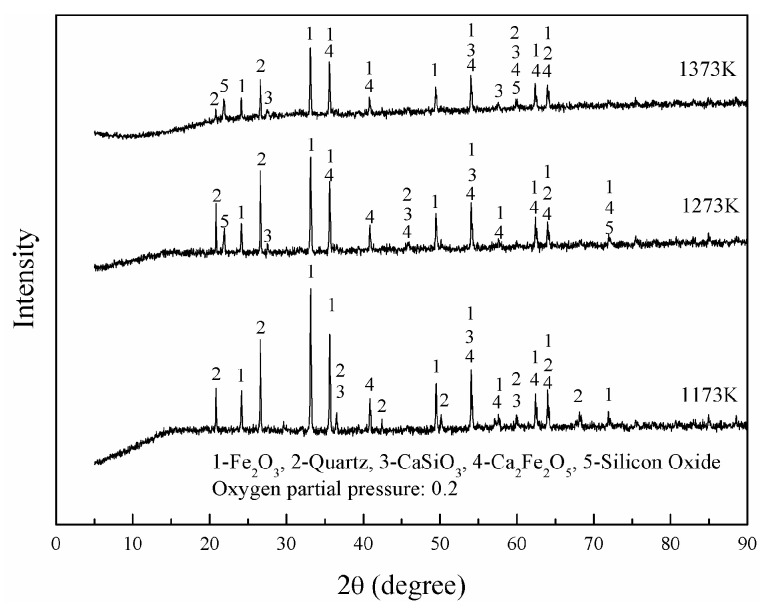
XRD results of calcines from Group A.

Figure 4 shows the change of nickel-volatilization percentage with roasting temperature. The volatilization percentages are enhanced with higher temperature. They increase from 31.4% to 58.1%, from 39.4% to 84.8%, and from 40.4% to 93.7% as the roasting temperature increases from 1173 K to 1373 K when the molar ratios of CaCl_2_ to NiO are 5, 7.5 and 10, respectively. Figure 5 shows the XRD results of calcines from Group A after roasting with the oxygen partial pressure of 0.2. There are newly generated CaSiO_3_ and CaFe_2_O_4_ and unreacted SiO_2_ and Fe_2_O_3_ in the NiO-SiO_2_-Fe_2_O_3_-CaCl_2_ system at the investigated temperature range with a molar ratio of 10 CaCl_2_ to NiO and a molar ratio of 4 SiO_2_ to CaCl_2_.

It is inevitable that part of the CaCl_2_ evaporates from the NiO-SiO_2_-Fe_2_O_3_-CaCl_2_ system at temperatures between 1173 K and 1373 K which are above the melting point of CaCl_2_ [16]. The reaction could be written as

CaCl_2_ = CaCl_2_ (g). (5)

Solid or liquid NiCl_2_ (melting point 1304 K) [17] is converted into a gaseous state at a higher temperature, which could be written as

NiCl_2_ = NiCl_2_ (g). (6)

**Figure 6:**
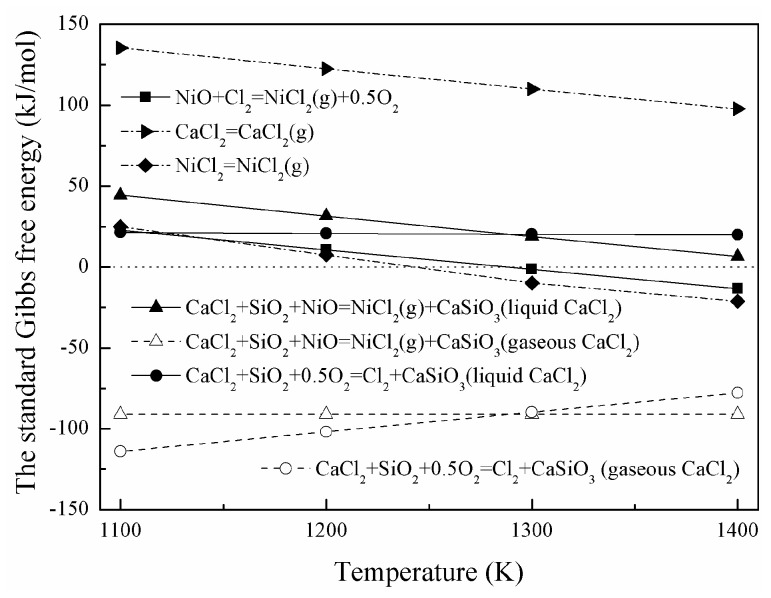
The standard Gibbs free-energy changes of high-temperature chlorination of nickel oxide.

The standard Gibbs free-energy changes of Equations (2)–(6) are calculated from the thermochemical data [17] and are shown in Figure 6. $\Delta G_{2}^{0}$ decreases significantly as the temperature increases and becomes closer to zero producing liquid CaCl_2_. Reaction (2) cannot happen in the standard state due to the positive value of $\Delta G_{2}^{0}$. However, Δ*G*_2_ could decrease and turn from positive to negative by decreasing the partial pressures of gaseous NiCl_2_ according to Equation (7). In this case, Reaction (2) can possibly occur. It is almost unchanged and significantly below zero in the investigated temperature range producing gaseous CaCl_2_. This indicates that a higher temperature could promote the direct chlorination of nickel oxide. $\Delta G_{3}^{0}$ decreases slightly, and all the values are less than 21.5 kJ/mol producing liquid CaCl_2_. Similarly, Reaction (3) can possibly occur when Δ*G*_3_ turns from positive to negative by decreasing the partial pressures of Cl_2_ according to Equation (8). Although it becomes significantly larger, all the values are much lower than zero producing gaseous CaCl_2_. Therefore, Reaction (3) could happen in the investigated temperature range. $\Delta G_{4}^{0}$ turns from positive to negative as temperature increases, which suggests that the chlorination of nickel oxide with Cl_2_ generated from the decomposition of CaCl_2_ is enhanced. Therefore, a higher temperature could improve the indirect chlorination of nickel oxide.

(7) $$\Delta G_{2}=\Delta G_{2}^{0}+RT\ln\frac{a_{{\mathrm{CaSiO}}_{3}}(P_{{\mathrm{NiCl}}_{2}}/P^{0})}{a_{{\mathrm{CaCl}}_{2}}a_{{\mathrm{SiO}}_{2}}a_{\mathrm{NiO}}},$$

(8) $$\Delta G_{3}=\Delta G_{3}^{0}+RT\ln\frac{a_{{\mathrm{CaSiO}}_{3}}(P_{{\mathrm{Cl}}_{2}}/P^{0})}{a_{{\mathrm{CaCl}}_{2}}a_{{\mathrm{SiO}}_{2}}{\left(P_{O_{2}}/P^{0}\right)}^{0.5}}.$$

**Figure 7:**
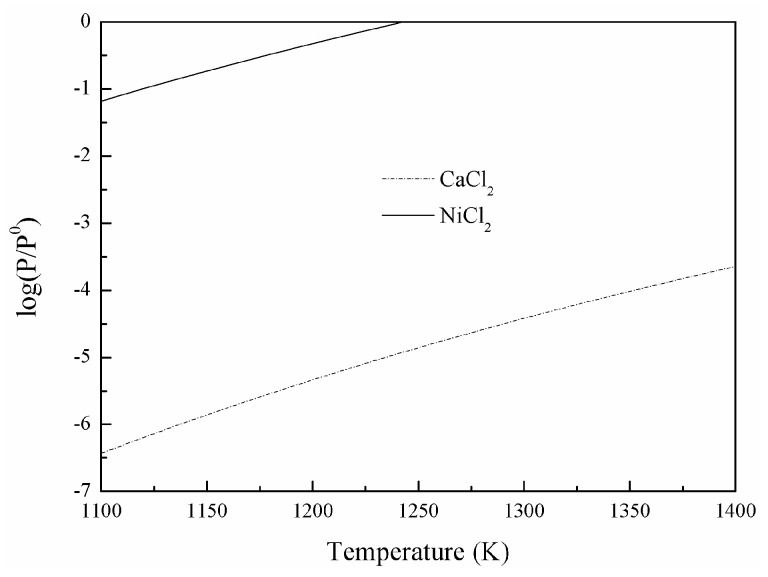
The change of saturated vapor pressures of NiCl_2_ and CaCl_2_ with temperature.

On the other hand, $\Delta G_{5}^{0}$ decreases significantly and $\Delta G_{6}^{0}$ turns from positive to negative as the temperature increases. This suggests that CaCl_2_ and NiCl_2_ tend to evaporate from the NiO-SiO_2_-Fe_2_O_3_-CaCl_2_ system at a higher temperature. Compared with liquid CaCl_2_, Reactions (2) and (3) are more achievable with gaseous CaCl_2_, as shown in Figure 6. Reactions (2) and (4) are promoted with the continuous gaseous NiCl_2_ volatilization from the system which breaks the reaction balances. The saturated vapor pressures of CaCl_2_ and NiCl_2_ are calculated and shown in Figure 7. They increase significantly with a rise of temperature, which leads to the enhancement of volatilization percentage as it increases the roasting temperature.

### 3.5. Effect of Oxygen Partial Pressure of Carrier Gas

**Figure 8:**
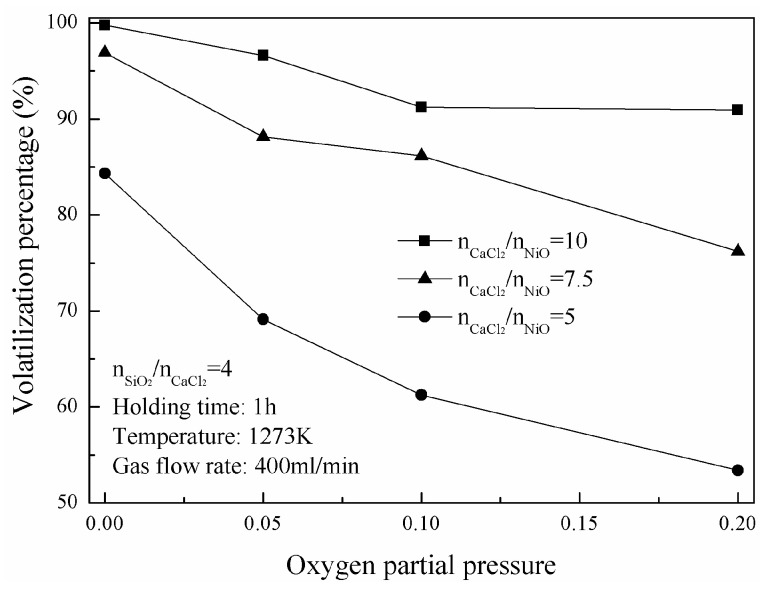
Change of nickel-volatilization percentage with oxygen partial pressure.

Nickel-volatilization percentage is significantly influenced by oxygen partial pressure. As shown in Figure 8, nickel-volatilization percentages decrease from 84.3% to 53.4% as increasing the oxygen partial pressure from near zero to 0.2 at 1273 K with the molar ratio of five CaCl_2_ to NiO. And, the percentages decrease from 96.9% to 76.1% and from 99.7% to 90.9% at 1273 K with the molar ratios of CaCl_2_ to NiO of 7.5 and 10, respectively. The magnitude of volatilization percentage reduction caused by increasing the oxygen partial pressure becomes narrower with a larger molar ratio of CaCl_2_ to NiO.

**Figure 9:**
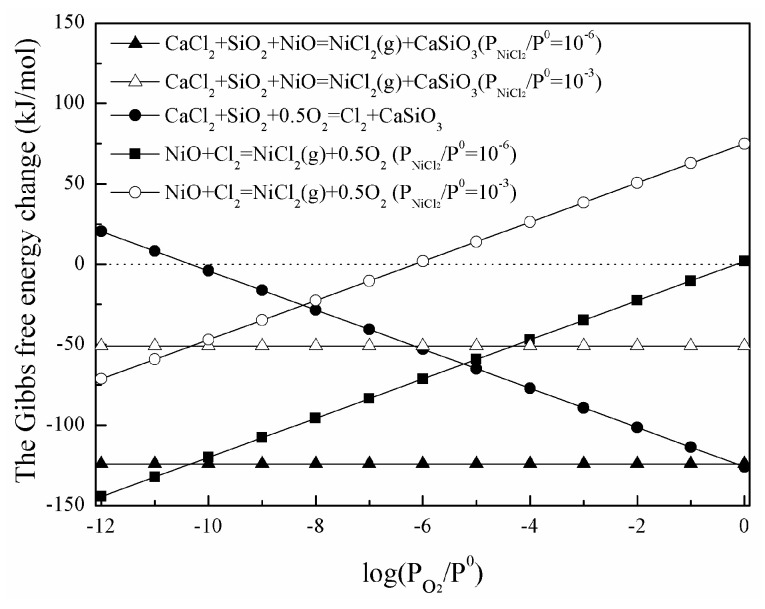
The Gibbs free-energy changes of reactions with different oxygen partial pressure.

Assuming that the activities of CaCl_2_, NiO, SiO_2,_ and CaSiO_3_ are taken together and Cl_2_ is equal to 10^−6^, the Gibbs free-energy changes of reactions with different oxygen partial pressure at 1273 K are calculated according to Equations (7)–(9) and shown in Figure 9. It should be noted that only the case of liquid CaCl_2_ is considered. It could be seen that the direct high-temperature chlorination of nickel oxide is not affected by oxygen partial pressure, but influenced by the NiCl_2_ partial pressure. Reaction (3) is promoted as increasing oxygen partial pressure, and Reaction (4) is inhibited with both greater oxygen and NiCl_2_ partial pressure.

(9) $$\Delta G_{4}=\Delta G_{4}^{0}+RT\ln\frac{(P_{{\mathrm{NiCl}}_{2}}/P^{0}){\left(P_{O_{2}}/P^{0}\right)}^{0.5}}{a_{\mathrm{NiO}}{\left(P_{{\mathrm{Cl}}_{2}}/P^{0}\right)}^{0.5}}.$$

**Figure 10:**
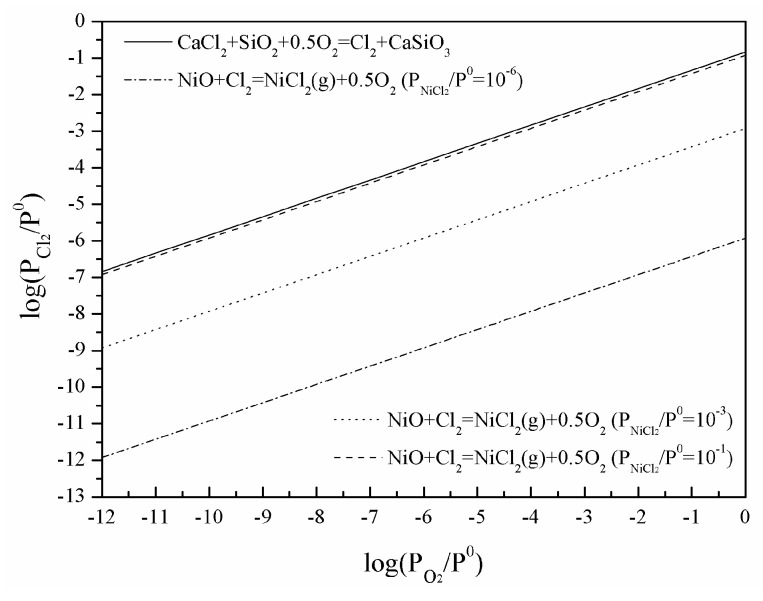
The equilibrated chlorine partial pressure at 1273 K.

The equilibrated chlorine partial pressure of Reactions (3) and (4) at 1273 K could be calculated using Equations (10) and (11), respectively, and shown in Figure 10, assuming that activities of CaCl_2_, NiO, SiO_2,_ and CaSiO_3_ are taken together. It could be seen that the equilibrated chlorine partial pressure calculated using Equation (10) is larger than that using Equation (11) at the same oxygen partial pressure with a smaller NiCl_2_ partial pressure only considering liquid CaCl_2_. This indicates that Cl_2_ generated with Reaction (3) could meet the chlorine-partial-pressure requirement of Reaction (4). Therefore, the indirect chlorination happens. The chlorine-partial-pressure demand of Reaction (4) enhances as NiCl_2_ partial pressure increases from 10^−6^ to 10^−1^ and gets close to the corresponding equilibrated chlorine partial pressure calculated with Equation (10). Indirect chlorination is inhibited in this case no matter the oxygen partial pressure.

(10) (PCl2/P0)Reaction(3)=aCaSiO3aCaCl2(PO2/P0)0.5aSiO2e−ΔG30RT,

(11) (PCl2/P0)Reaction(4)=(PNiCl2/P0)(PO2/P0)0.5aNiOeΔG40RT.

To sum up, when oxygen partial pressure is close to zero, Reaction (3) does not happen and there is only direct high-temperature chlorination. As the oxygen partial pressure increases, Reaction (3) starts to occur. However, NiCl_2_ partial pressure keeps at a high level as a result of the direct high-temperature chlorination, which leads to the inhibition of Reaction (4). Cl_2_ generated using Reaction (3) could not meet the chlorine partial pressure requirement of Reaction (4), and part of CaCl_2_ is consumed and the volatilization percentage decreases. A further increase of oxygen partial pressure leads to a rising chlorine partial pressure which meets the demand of Reaction (4). The indirect high-temperature chlorination happens. When oxygen partial pressure is large enough, Reaction (4) is inhibited. In this condition, the generation rate of Cl_2_ using Reaction (3) increases gradually, and becomes greater than the chlorination rate of nickel oxide. Excess Cl_2_ escapes from the system leading to the lower utilization efficiency of CaCl_2_. Therefore, nickel-volatilization percentage is negatively correlated to oxygen partial pressure. With a larger molar ratio of CaCl_2_ to NiO, there is still enough CaCl_2_ used for the direct high-temperature chlorination, leading to a narrower magnitude of volatilization-percentage reduction caused by oxygen partial pressure.

### 3.6. High-Temperature-Chlorination Kinetics

The reaction process of the high-temperature chlorination of nickel oxide using calcium chloride is extremely complicated. In the case of direct chlorination, the process mainly consists of the phase transformation of CaCl_2_ from liquid to gas phase, the chlorination reaction between nickel oxide and liquid or gaseous CaCl_2_, and the volatilization of NiCl_2_. Indirect chlorination is unavoidable due to the existence of trace amounts of oxygen. The side-reaction process mainly includes the phase transformation of CaCl_2_ from liquid to gas phase, the reaction between trace amounts of oxygen and liquid or gaseous CaCl_2_ to give off Cl_2_, the chlorination reaction between nickel oxide and Cl_2_, and the volatilization of NiCl_2_. The macro-kinetics of high-temperature chlorination of nickel oxide using calcium chloride is established according to the unreacted shrinking-core model.

For the chemical-reaction control model, the relationship between nickel-volatilization percentage and time could be expressed as [18]

[1 − (1 − X)^1/3^] = kt. (12)

For the diffusion control model, the relationship could be written as [18]

[1 − 3(1 − X)^2/3^ + 2(1 − X)] = kt, (13)

where X is the nickel-volatilization percentage; k is the apparent reaction rate constant; and t is time.

**Figure 11:**
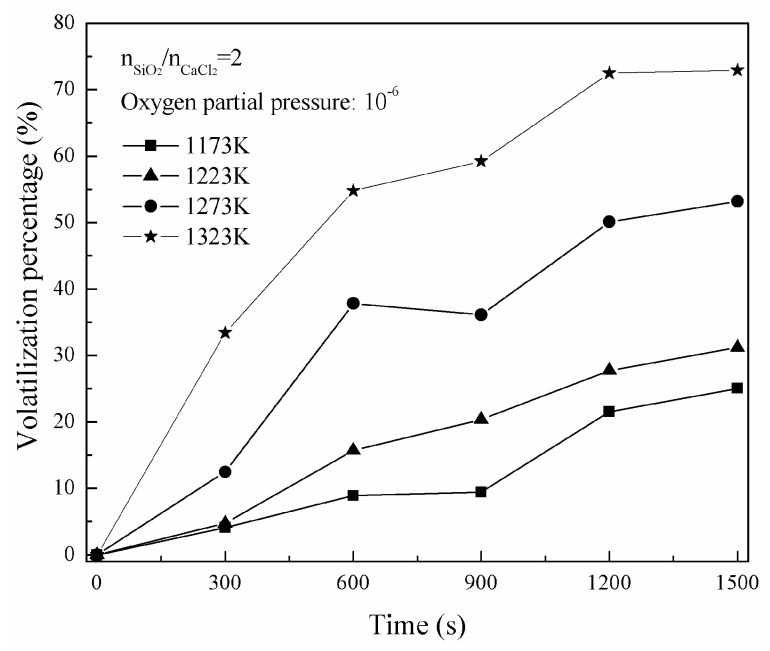
Change of nickel-volatilization percentage with time.

**Figure 12:**
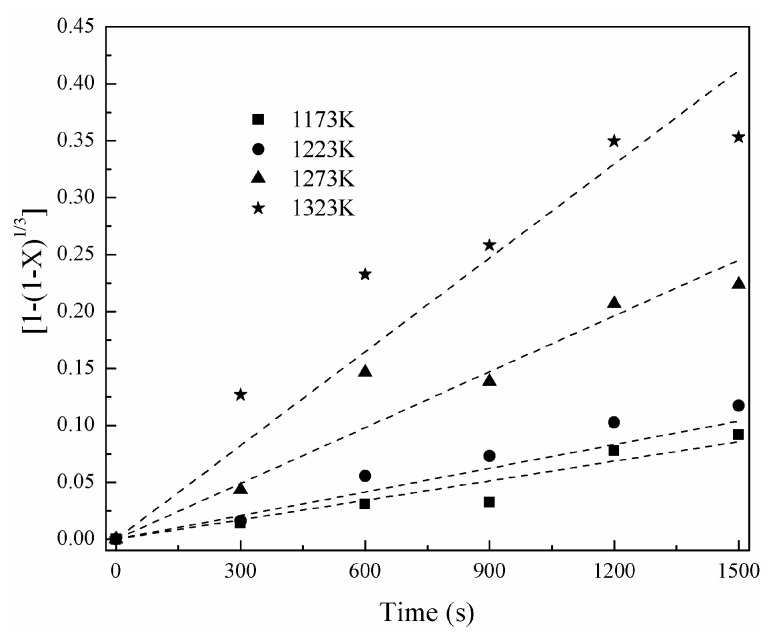
Relationship between [1 − (1 − X)^1/3^] and time.

**Figure 13:**
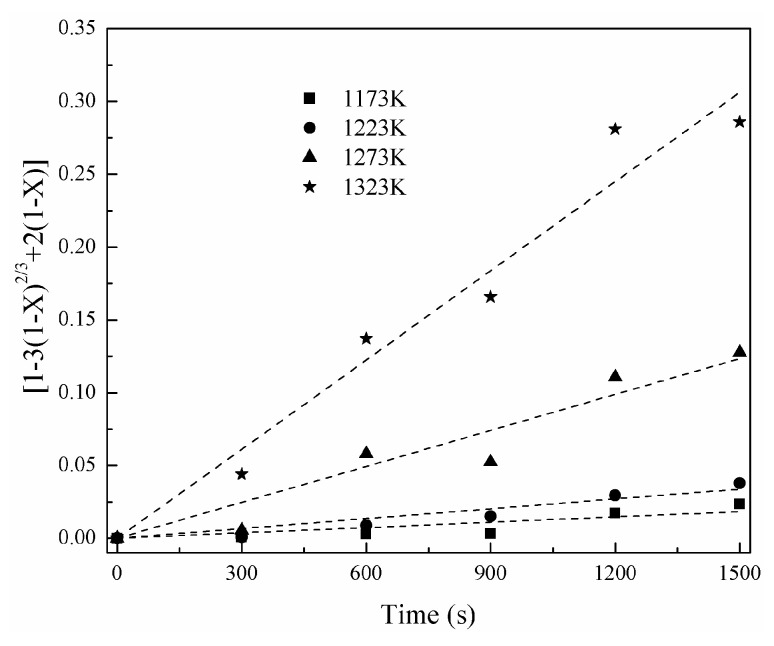
Relationship between [1 − 3(1 − X)^2/3^ + 2(1 − X)] and time.

**Figure 14:**
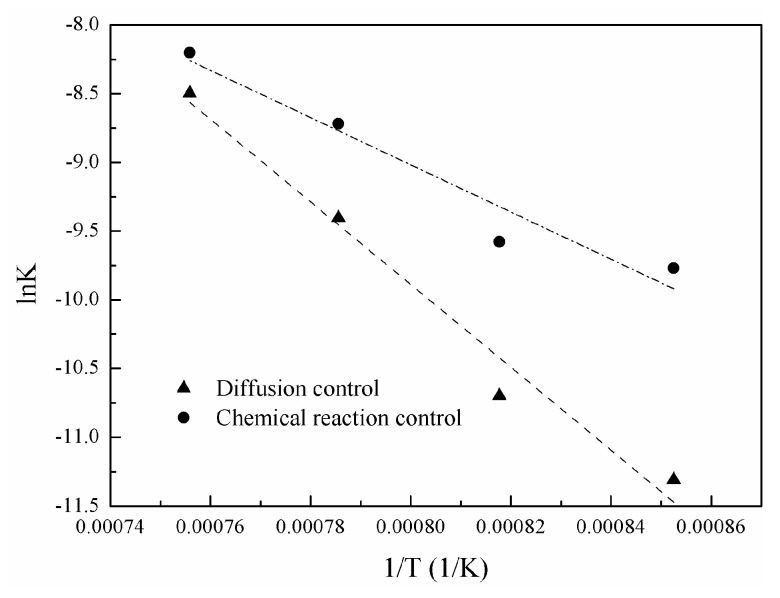
Relationship between lnK and 1/T.

Figure 11 shows the change of nickel-volatilization percentage with time. According to Equation (12), a change of [1 − (1 − X)^1/3^] with time is obtained and shown in Figure 12. Similarly, a change of [1 − 3(1 − X)^2/3^+2(1 − X)] with time is also obtained according to Equation (13) and shown in Figure 13. The apparent reaction-rate constants at different temperatures are calculated to the straight slopes in Figure 12 and Figure 13 for the chemical-reaction control model and diffusion control model, respectively, and are shown in Figure 14. According to the Arrhenius law, the apparent activation energies are calculated as 142.91 kJ/mol and 250.22 kJ/mol for the chemical-reaction control model and diffusion control model, respectively.

The activation energies from 423 to 473 K and from 473 to 673 K were 46.1 and 12.4 kJ/mol for the chlorination of nickel oxide with gaseous hydrogen chloride, respectively [14]. And, it was 12.4 kJ/mol for the chlorination of nickel-containing lateritic iron ore with gaseous hydrogen chloride [15]. The values suggest that the chlorination of nickel oxide with gaseous hydrogen chloride was controlled by the diffusion of gaseous reactant [14,15]. The chlorination of hematite with Cl_2_ is probably diffusion controlled between 873 K and 1148 K with the activation energy of 74 kJ/mol [19].

The activation energy was determined to be 119 kJ/mol, which confirmed the assumption that the chlorination of NiFe_2_O_4_ with Cl_2_ between 973 K and 1173 K was chemically controlled [6]. The apparent activation energies of the PbSO_4_ chlorination and carbochlorination were about 174 kJ/mol and 114 kJ/mol, respectively, which suggested that both the rates of chlorination and carbochlorination were probably controlled by the chemical reaction [20].

Based on the above analysis, the activation energy of diffusion control is lower than that of chemical-reaction control. Therefore, such values of apparent activation energies suggest that it is more likely controlled by the chemical reaction of high-temperature chlorination of nickel oxide using calcium chloride.

## 4. Conclusions

The conclusions obtained from this study are listed as follows:

(1) Nickel-volatilization percentage increases as gas-flow rates increase from 0 to 200 mL/min, and then is kept almost at a constant with a higher gas-flow rate and greater CaCl_2_ dosage.

(2) Nickel-volatilization percentage is positively correlated to temperature from 1173 K to 1373 K and to CaCl_2_ dosage.

(3) Nickel-volatilization percentage is negatively correlated to oxygen partial pressure.

(4) The high-temperature chlorination of nickel oxide using calcium chloride is controlled by a chemical reaction. The apparent activation energy is 142.91 kJ/mol and between 1173 K and 1323 K.

## Figures and Tables

**Table 1:** Sample compositions and CaCl_2_ dosage.

Group	NiO (g)	SiO_2_ (g)	Fe_2_O_3_ (g)	CaCl_2_ (g)
A	1	32.18	66.82	14.86
B	1.33	32.18	66.49	14.86
C	2	32.18	65.82	14.86
D	1.33	16.09	82.58	14.86

## Data Availability

The datasets analyzed or generated during the study are available from the corresponding author on reasonable request.
